# Treatment-Based Strategy for the Management of Post-Kala-Azar Dermal Leishmaniasis Patients in the Sudan

**DOI:** 10.1155/2013/708391

**Published:** 2013-04-15

**Authors:** A. M. Musa, E. A. G. Khalil, B. M. Younis, M. E. E. Elfaki, M. Y. Elamin, A. O. A. Adam, H. A. A. Mohamed, M. M. M. Dafalla, A. A. Abuzaid, A. M. El-Hassan

**Affiliations:** ^1^The Leishmaniasis Research Group, Institute of Endemic Diseases, University of Khartoum, Sudan; ^2^Department of Clinical Pathology and Immunology, Institute of Endemic Diseases, University of Khartoum, P.O. Box 102, Sudan

## Abstract

Post-kala-azar dermal leishmaniasis (PKDL) is a dermatosis that affects more than 50% of successfully treated visceral leishmaniasis (VL) patients in Sudan. PKDL is considered an important reservoir for the parasite and its treatment may help in the control of VL. Currently, treatment is mainly with sodium stibogluconate (SSG), an expensive and fairly toxic drug and without universally in treatment protocols used. A literature review, a consensus of a panel of experts, and unpublished data formed the basis for the development of guidelines for the treatment of PKDL in the Sudan. Six treatment modalities were evaluated. Experts were asked to justify their choices based on their experience regarding of drug safety, efficacy, availability, and cost. The consensus was defined by assigning a categorical rank (first line, second line, third line) to each option. Regarding the use of AmBisome the presence of the drug in the skin was confirmed in smears from PKDL lesions. Recommendations: AmBisome at 2.5 mg/kg/day/20 days or SSG at 20 mg/kg/day/40 days plus four/weekly intradermal injection of alum-precipitated autoclave *L. major* vaccine are suggested as first- and second-treatment options for PKDL in the Sudan, respectively. SSG at 20 mg/Kg/day/60 or more days can be used if other options are not available.

## 1. Introduction

Post-kala-azar dermal Leishmaniasis (PKDL) is a complication of visceral Leishmaniasis (VL) that emerges as a new disease entity following successful treatment of VL. In few cases PKDL may follow subclinical infection with *L donovani*. It occurs with a frequency of ~56% of successfully treated VL patients in the Sudan [1–5]. The disease has been described with a lower frequency in India, Nepal, and Bangladesh [6]. In Sudan, the skin lesions heal spontaneously in ~85% of patients, while in 15% of patients lesions persist and require medical treatment [4]. The disease can be very severe affecting the mucous membrane and interfering with feeding in the very young [4]. PKDL lesions (especially nodular forms) are probably an important source of transmission in the Sudan and the Indian subcontinent [7]. In addition, some reports suggested that the incidence of antimony refractoriness in VL patients is due to the anthroponotic transmission of refractory strains from PKDL patients thus increasing the burden of drug resistance [8].

Cytokines, drugs, and Ultraviolet light (UVB) have been suggested as possible factors involved in the pathogenesis of Sudanese PKDL [9–11]. The pathology is well documented. It consists of hyperkeratosis, parakeratosis, acanthosis, follicular plugging, and liquefaction degeneration of the basal layer of the epidermis. In the dermis, there are varying intensities of inflammation with scanty parasites associated with an infiltrate composed of lymphocytes, macrophages, and epithelioid cells. Discrete granulomas may be found in some patients [3].PKDL skin lesions mimic many dermatological conditions. Miliaria rubra was the most common while leprosy was the most challenging differential diagnosis [3]. Detection of *L. donovani* parasites in the skin confirms the diagnosis of PKDL but these are difficult to find in routine sections and smears. Molecular techniques could provide a way out, but these are expensive and are usually not available under field conditions. Rash appearance spatial relationship to VL treatment, intact sensations, antileishmania positive serology and histopathology can provide good alternative diagnostic criteria [3, 12–15]. Positive serology is limited by the fact that it can be due to previous VL. Persistence of the lesions is frequently associated with nonreactivity in the Leishmanin skin test (LST) and high levels of anti-Leishmanial antibodies [4].

PKDL patients with reactive LST have a favorable prognosis; lesions either heal spontaneously or respond better to chemotherapy [4, 5, 16]. Sodium stibogluconate (SSG) is currently the treatment of choice for severe and persistent PKDL in Sudan. Treatment requires prolonged hospital stay (>60 days) for daily intravenous or painful intramuscular SSG injections. The drug has a number of toxicities ranging from arthralgia to cardiac toxicities and acute pancreatitis; in addition, drug unresponsiveness is mounting especially in the Indian subcontinent [3, 17–21].

Sodium stibogluconate in combination with allopurinol and/or rifampicin and immunomodulators were not shown to be advantageous in small numbers of patients in India [22]. Azoles in forms of ketoconazole and a combination of itraconazole and terbinafine were not efficacious [1, 23]. Miltefosine 150 mg/day for 60 days or 100 mg/day for 90 days produced initial cure rate 96%, but the duration of treatment had to be reduced due to gastrointestinal symptoms [24]. In HIV patients, PKDL was successfully treated with Miltefosine with remissions for 3–6 months [25].

It has been documented in a number of studies that AmBisome is a suitable drug for treatment of PKDL ([26, 28], unpublished data). In Sudan, the cure rate with immunochemotherapy was significantly better than with chemotherapy alone. The vaccine used consisted of a mixture of killed *L. major* adsorbed onto alum plus Bacillus Calmette-Guérin (BCG), given four times at weekly intervals plus SSG, at 20 mg/kg/day for 40 days [16, 27].

In this paper, published and unpublished data, and opinions of researchers with experience in PKDL management were asked to formulate evidence-based guidelines for the treatment of PKDL in the Sudan.

## 2. Materials and Methods

### 2.1. Options for Treatment of PKDL

Published data from Sudan and unpublished data on PKDL management, a consensus of a panel of 7 Sudanese clinicians and researchers with formed the basis for the development of an evidence-based guide for the treatment of post-kala-azar dermal Leishmaniasis. The experts were selected based on their experience treating VL and PKDL using available drugs in common use in the Sudan. Some of them have been treating VL and PKDL for the last 30 years. For the consensus, a 6-question survey was developed with 3 options per question (1: poor option; 2: good option; 3: best option) with six treatment modalities included in survey. For each treatment modality, the experts also commented on the drug safety, efficacy, availability, and cost. Experts were asked to justify their choices based on their experience. The consensus was defined by assigning a categorical rank (first-line/preferred, second-line/first alternative, third-line/second alternative) to each option. 

### 2.2. Detection of AmBisome in PKDL Lesions

To prove that AmBisome does reach the skin and is delivered to parasitized macrophages, we prepared sections from a heavily infected patient and traced AmBisome in his skin. A smear from the lesion was heat-fixed and stained with Sudan Black for lipids. As control, a smear from the lesion of the patient before treatment was treated in the same way as the smear during AmBisome treatment.

## 3. Results

### 3.1. Consensus of Panel of Experts

AmBisome was considered to be the first option. This was justified by high cure rate, short hospital stay and negligible side effects. AmBisome, even after reduction of the cost, remains relatively expensive as it is used for 20 days. This is followed as a second choice by immunochemotherapy since it reduces the cost of treatment, may prevent drug-resistance but the logistics involved in vaccine importation from abroad or making it locally make it inferior to AmBisome. Although sodium stibogluconate is toxic and treatment takes a long time, it can be used in the absence of these two options. The azoles were considered ineffective and Amphotericin-B was regarded to be very toxic.

### 3.2. Results of the presence and distribution of AmBisome in PKDL Lesions

**Figure 1:**
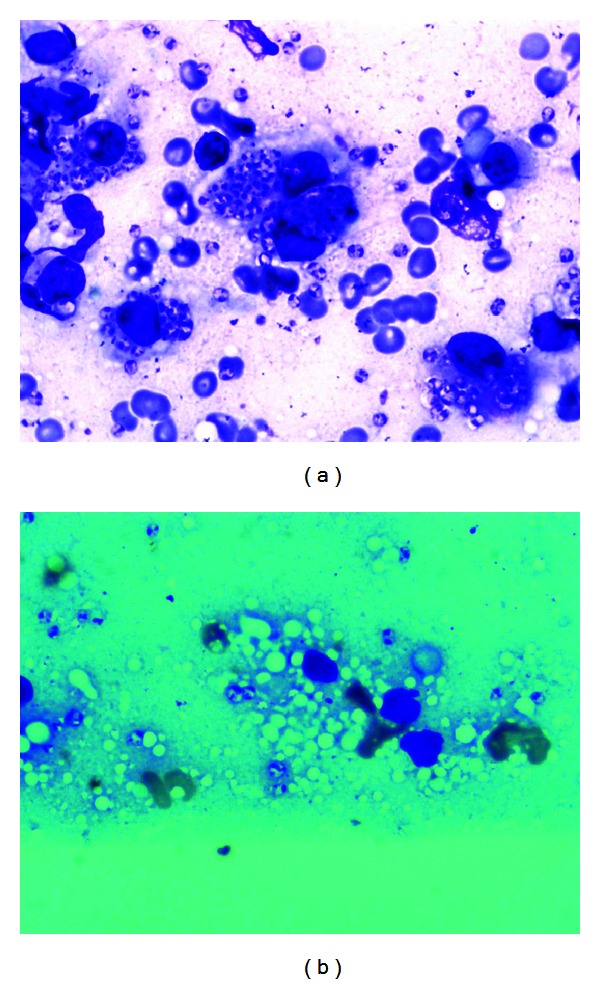
(a) Skin lesion before AmBisome. Heavy parasites in macrophages. No vacuoles. (b) Skin lesion during AmBisome. Vacuoles inside macrophages where the lipid of AmBisome was after heat fixation in alcohol. Parasites are scanty.

Figure 1(a) shows heavily parasitized macrophages before AmBisome treatment.  Figure 1(b) shows vacuoles inside macrophages where the lipid of AmBisome was demonstrated after heat fixation of the smear. Parasites are scanty. 

## 4. Discussion

In Sudan, control of visceral Leishmaniasis was suggested to be dependent on control of PKDL [7]. This was proved already in India [29]. It was documented by Thakur and colleagues that reduction in the incidence of PKDL followed the use of Amphotericin B for the treatment of VL [29]. In a clinical trial conducted recently in East Africa to evaluate the combination of SSG and paromomycin (PM) as a first line treatment for VL it was reported that PKDL incidence was reduced by 50% among the SSG and PM treated group compared to the SSG group [30]. The limitation of this study was that individuals were followed for 6 months only after treatment of VL, but further followup confirmed this outcome [Musa et al., under preparation]. Although the new combination showed reduction of the incidence by 50%, but it is difficult to extrapolate data to comment on its efficacy on PKDL. Although SSG is available, its toxicity, cost, and lengthy hospital stay have hampered its use as the first choice for treatment PKDL in Sudan. Its efficacy is related to prolonged treatment which may add to its toxicity and cost [1]. As a continuing effort of the Leishmaniasis Research Group/Sudan, Institute of Endemic Diseases, to find less costly, less toxic, and efficacious treatment PKDL, the use of Azoles, AmBisome, and immunochemotherapy were studied. The fact that Azoles (either as monotherapy or in combination) are attractive options because they can be taken orally had received much attention early on. Unfortunately, they did not show any promise [1, 23]. This failure is probably due to the fact that *L. donovani* survives the toxicity of azoles by using sterols from the host cells [1, 23]. AmBisome proved to be safe and efficacious [26, 28]. It reaches the skin in which it is engulfed by the macrophages where the *Leishmania* parasites reside. Its action is directly on the cell membrane of the parasites (Figures 1(a) and 1(b)). Although AmBisome is expensive but its high efficacy, the relatively short duration of treatment, the short hospital stay, and the negligible side effects makes it a useful first line drug to use (see Table 1).

Immunochemotherapy has proven effective in healing the lesions of persistent PKDL cases in a relatively shorter duration compared to SSG alone [16]. It was tried on the background that the pathogenesis of PKDL is immunologically mediated. The immunochemotherapy accelerates healing by shifting the immune response from Th2 or Th1/Th2 to Th1 as evidenced by conversion in Leishmanin skin reaction and production of high IFN-*γ* and low IL-10 in peripheral blood mononuclear cells stimulated by *Leishmania* antigens [16]. With all the problems involved in vaccine importation from abroad or in making it locally, immunochemotherapy cannot be the best option for now. Unpublished data from Sudan showed that Amphotericin B is very toxic, intolerable, and difficult to handle (personal communication). In view of high cost of AmBisome, and the logistics of making the vaccine available and failure of other treatment options, an alternative of combining SSG with paromomycin, which is a cheap and readily available drug is important, but it is not certain whether this combination will be of promise to treat PKDL lesions within a reasonable treatment duration.

## 5. Recommendations

AmBisome at a dose of 2.5 mg/kg/day intravenously for 20 days is safe and can markedly reduce hospital stay. It is therefore considered a first-line treatment. Intravenous or intramuscular SSG at a dose of 20 mg/kg/day for 40 days plus weekly intradermal injection of alum-precipitated autoclave *Leishmania major* vaccine for 4 weeks is suggested as the a second form of treatment. Alternatively, 60 or more days of SSG after complete cardiovascular and liver function assessment can be the last resort. This is in line with the recommendation made by the WHO expert committee of PKDL treatment in East Africa with minor modification [31].

## Figures and Tables

**Table 1:** Published and unpublished data on PKDL in Sudan.

Treatment	Dose/duration	Number of patients	Efficacy	Safety	Total cost^1^	Reference	Route
Published studies							
Pentostam	20 mg/kg/d/30–60 d	>100	Efficacious	++	US$249.6–499.2	[1]	im/iv
Ketoconazole	10 mg/kg/d/30 d	>20	Not efficacious	+	US$55.00	[1]	oral
Itraconazole and	200 mg/d/30 d	9	Not efficacious	+	US$250	[23]	oral
Terbinafine	250 mg/d/30 d						
AmBisome	3 mg/kg/d/30 d	2	Efficacious	Safe	US$1,350	[26]	iv
AmBisome	2.5 mg/kg/d/ 20 d	12	Efficacious	Safe	US$756	[28]	iv
Immunochemotherapy	20 mg/kg/d/40 d (SSG) (wkly id Alum-ALM dose/4 weeks)	35	Efficacious	+	US$100.0	[16, 27]	iv/im
Unpublished studies							
AmBisome	2.5 mg/kg/d/ 20 d	27	Efficacious	Safe	US$756	Khalil et al.	
SSG	20 mg/kg/d/60 d	>100	Efficacious	++	US$121.03	Khalil and Musa	
Amphotericin B	0.5 mg/kg/d/ 30 d	7	Efficacious	+++	US$90	Musa et al.	

SSG: sodium stibogluconate; d: day; wk: week. Alum-ALM: alum-precipitated autoclaved *L. major* plus BCG.

Toxicity graded as: +: acceptable side effects; ++: moderately toxic; +++: highly toxic.

^1^The total cost was calculated for a patient of 40 kg; almost all expenses are the same for a single patient regardless of the regimen except the cost of drug. The unit prices of injectable drugs and oral drugs were obtained from the WHO records and National Medicines and Poisons Board (NMPB, Sudan), respectively.
